# Distribution Patterns and Community Assembly of Soil Nematodes Along Elevation Gradients in a Dry‐Hot Valley

**DOI:** 10.1002/ece3.74358

**Published:** 2026-09-30

**Authors:** Jinxiu Zhang, Haoran Lei, Na Lin, Chunyu Hou, Chunyu Yue, Yajun Chen, Jianping Wu

**Affiliations:** ^1^ State Key Laboratory of Vegetation Structure, Function and Construction (VegLab), Yunnan Key Laboratory of Plant Reproductive Adaptation and Evolutionary Ecology, School of Ecology and Environmental Science Yunnan University Kunming China; ^2^ School of Geography and Remote Sensing Ningbo University Ningbo China; ^3^ CAS Key Laboratory of Tropical Forest Ecology, Xishuangbanna Tropical Botanical Garden Chinese Academy of Sciences Mengla China; ^4^ Yuanjiang Savanna Ecosystem Research Station, Xishuangbanna Tropical Botanical Garden Chinese Academy of Sciences Yuanjiang China

**Keywords:** arid ecosystems, community assembly, distribution pattern, elevation gradients, soil nematode

## Abstract

In arid regions, mountain ecosystems are biodiversity hotspots but also highly fragile. While aboveground communities have been well studied, belowground communities—particularly soil fauna—remain poorly understood despite their key roles in ecosystem functioning. Soil nematodes play an important role in soil faunal communities. Here, we characterized soil nematode communities and soil properties in plots located at four different elevations in the Yuanjiang dry‐hot valley, China. We examined the effects of soil properties on nematode abundance, diversity, and community composition, and explored the community assembly along the elevational gradient. Our findings suggest that the increase in soil moisture is the primary driver of the elevational rise in nematode abundance, but it is not the main factor responsible for changes in nematode diversity. Instead, with increasing elevation, NH_4_^+^‐N simultaneously promoted the abundance and diversity of plant parasites nematodes while reducing the diversity of bacterivores nematodes. We also found that NH_4_^+^‐N and MBC exerted stronger influences on nematode community composition than soil moisture, underscoring the importance of nutrient‐related factors in shaping belowground biodiversity. Furthermore, soil moisture does not influence soil nematode community assembly as an environmental filter, but rather indirectly affects community assembly by regulating nematode abundance, suggesting that nematode communities in this arid region are adapted to low‐moisture conditions. Our study provides new insights into the elevational distribution patterns of soil nematodes in arid mountain ecosystems and helps to fill a critical knowledge gap regarding nematode community assembly in arid environments.

## Graphical abstract

In the Yuanjiang dry‐hot valley, soil moisture is the primary driver of nematode abundance. Soil nutrients play a more important role in determining nematode diversity and community composition. Drought stress does not influence the assembly of soil nematode communities.

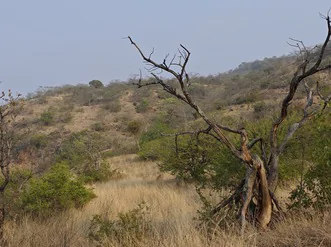

## 1 Introduction

Arid ecosystems are highly vulnerable to climate change due to poor soil fertility and sparse vegetation (Bradford et al. 2020). At the same time, climate models predict that the frequency, intensity, and geographic extent of droughts will continue to increase across the globe (Masson‐Delmotte et al. 2021). This trend underscores the growing importance of understanding the interactions among organisms inhabiting drought ecosystems. Mountain ecosystems serve as critical centers of biodiversity and ecological functionality in arid and semi‐arid regions (Qianwen et al. 2022). However, they also exhibit pronounced ecological fragility, making recovery from disturbances particularly difficult (Arif et al. 2023). Therefore, the distribution of biodiversity along elevational gradients has long been a central focus in ecological research (Rahbek et al. 2019). While much attention has been paid to aboveground organisms, belowground biodiversity remains comparatively understudied (Guerra et al. 2021). This gap is particularly evident in arid and semi‐arid environments, where investigations into the elevational patterns of soil biological communities are still scarce (Chen et al. 2024). However, soil organisms, particularly soil fauna, harbor remarkable diversity and play critical roles in ecosystem functioning (Anthony et al. 2023). Therefore, a deeper understanding of soil fauna in mountain ecosystems of arid regions is urgently needed.

Within soil faunal communities, soil nematodes exhibit remarkable diversity and play an important role within soil faunal communities (Anthony et al. 2023). Nematodes are grouped according to their feeding habits and are classified as bacterivores (BF), fungivores (FF), plant parasites (PP), and omnivore‐predators (OP) (Yeates et al. 1993). Due to these differences in feeding habits, soil nematodes occupy multiple trophic levels, thereby modulating food webs in ways that influence the entire soil biological community (de Ruiter et al. 1995; Ferris et al. 2001). More importantly, soil nematode communities have a substantial impact on terrestrial ecosystem processes, such as soil carbon and nitrogen cycling (Hunt and Wall 2002; Krumins et al. 2013). Elevation is considered a major factor influencing the abundance and diversity of nematode communities, as it encompasses substantial variation in climatic conditions (e.g., temperature and precipitation) and vegetation types (Afzal et al. 2021). However, existing studies report inconsistent patterns in nematode diversity and abundance along elevation gradients. Some studies suggest that nematode abundance and diversity decline with increasing elevation (Kashyap et al. 2022; Choudhary et al. 2023), whereas others report an increase along elevation gradients (Kouser et al. 2021). Additionally, several studies have observed a mid‐elevation peak in nematode abundance and diversity, indicating a unimodal pattern (Kergunteuil et al. 2016; Dong et al. 2017). At the same time, few studies have specifically focused on mountain ecosystems in arid regions; thus, the distribution patterns and drivers of nematode diversity and abundance in mountainous environments, particularly under arid conditions, remain to be further explored.

Community assembly research is essential for explaining species coexistence and the maintenance of biodiversity (Zhou and Ning 2017). In community ecology, two fundamental types of processes—deterministic and stochastic—govern community assembly patterns (Vellend 2010). Stochastic processes, including random birth, death, migration, dispersal, and colonization events, lead to community dynamics that are inherently unpredictable and largely independent of environmental conditions (Bell 2001; Rosindell et al. 2011). In contrast, deterministic processes involve predictable forces such as environmental filtering, interspecific competition, and predation, where shifts in community composition are driven by identifiable environmental gradients or biotic interactions (Chase and Leibold 2003; Gilbert 2012). Therefore, understanding the mechanisms of community assembly is crucial for explaining shifts in community composition in response to environmental change (Schlägel et al. 2020). Recent studies indicate that the relative importance of deterministic and stochastic processes in shaping soil nematode community assembly varies with geographic scale, soil environmental conditions, and organismal groups (Li et al. 2014; Moroenyane et al. 2016; Hou et al. 2022). Hence, it is necessary to investigate nematode community assembly across broader spatial scales (Pan et al. 2024). However, research specifically addressing the assembly processes of nematode communities in arid mountain ecosystems remains largely lacking.

In this study, we examined the abundance, diversity, community composition, and assembly mechanisms of soil nematodes along an elevational gradient in the Yuanjiang Dry‐Hot Valley, a savanna ecosystem located in southwestern China. This region is characterized by an arid climate that results from a unique combination of subsidence airflow and the Foehn effect (Jing 1999). More importantly, the climate in this valley is also strongly influenced by elevation. In the Yuanjiang Dry‐Hot Valley, both precipitation and soil moisture exhibit significant increases with elevation (Li et al. 2024), while soil nematode abundance, diversity, and community structure are known to be strongly affected by soil moisture availability (Bakonyi and Nagy 2000; Xiong et al. 2020; Bristol et al. 2023). In addition, other soil physicochemical properties, such as pH, organic matter content, and nutrient availability, also play important roles in nematode diversity (Zhou et al. 2022; Goncharov et al. 2023; Shao et al. 2023). Previous studies have shown that under strong environmental stress, soil nematode community assembly is predominantly governed by deterministic processes (Li et al. 2014; Moroenyane et al. 2016; Hou et al. 2022). Given the pronounced drought stress in our study region, we aimed to investigate the distribution patterns, driving factors, and assembly processes of soil nematode communities in arid mountain ecosystems. We hypothesized that (1) stochasticity in community assembly would increase with elevation as drought stress weakens, and (2) soil moisture and nutrients are key determinants of nematode abundance, diversity, and community composition. Our findings contribute novel insights into elevational patterns of soil nematodes in arid mountains and address a critical gap in understanding community assembly in dryland environments.

## 2 Materials and Methods

### 2.1 Study Sites

The study area is located within the Yuanjiang Dry‐Hot Valley Ecological Station in Yuanjiang County, Yunnan Province, China (101°21′24″–102°21′12″ E, 23°19′12″–23°46′12″ N). The Yuanjiang dry‐hot valley features a pronounced elevational gradient. At lower elevations, the combined effects of Foehn winds and subsiding air currents result in low soil moisture levels, and the vegetation is dominated by typical savanna ecosystems. Dominant plant species in the area include *Heteropogon contortus* , *Euphorbia royleana* , and *Woodfordia fruticosa*. In contrast, higher elevations are characterized by relatively intact montane evergreen broadleaf forests.

### 2.2 Soil Sampling

To investigate the distribution of nematode communities along the valley's elevation gradient, we collected soil samples on October 2–3, 2023 and January 1–2, 2024 near the valley's ecological station at elevations ranging between 400 and 1600 m. We established four 10 × 10 m plots in randomly selected locations in relatively flat areas at 400, 800, 1200, and 1600 m. Plots were separated by a distance of at least 30 m. Five samples were collected from the top 10 cm of soil in each plot, after which they were thoroughly mixed to form a single composite sample, resulting in 32 soil samples (4 elevations × 4 replicates × sampling times). Soil nematodes were extracted immediately from fresh soil, with the remaining soil reserved for physicochemical analysis.

### 2.3 Nematode Extraction and Identification

Nematode samples were extracted from fresh soil using the Baermann funnel method (Gray 1984). After removing roots and rocks, 50.0 g of fresh soil was transferred to a Baermann funnel, where it was mixed with pure water for nematode extraction over a period of 48 h. Only live nematodes that remained active in the soil were extracted from the soil, after which they were quantified using a counting plate and a microscope.

Microscopic glass slides were prepared from each sample for nematode identification. 100 nematodes (or all nematodes in samples containing fewer than 100) were selected at random from each sample. Genus was then determined for each individual based on morphological characteristics. Both nematode counting and identification were conducted using a Leica microscope (DM 3000). Following identification, nematodes were classified into four feeding groups: BF, FF, PP, or OP (Yeates et al. 1993). Finally, Shannon diversity was calculated as an indicator of nematode diversity (Shannon 1948).

### 2.4 Soil Physicochemical Analysis

After passing soil through a 2 mm sieve, a portion was reserved to determine ammonium nitrogen (NH_4_^+^‐N), nitrate nitrogen (NO_3_^−^‐N), soil moisture, microbial biomass carbon (MBC), and dissolved organic carbon (DOC). Remaining soil was air‐dried, with a portion finely ground and passed through a 0.85 mm sieve to measure available phosphorus (AP) and pH. Another portion of the air‐dried soil was finely ground and passed through a 0.15 mm sieve to determine total nitrogen (TN), total phosphorus (TP), and soil organic carbon (SOC). NH_4_^+^‐N, NO_3_^−^‐N, TN, TP, and AP were analyzed using an automatic discrete chemical analyzer (Clever Chem 380, Germany). Soil moisture and pH were measured using standard methods (Dinnis 1994). MBC was extracted using chloroform fumigation, and MBC, SOC, and DOC were analyzed using an automatic TOC analyzer (Elementar, Germany).

### 2.5 Data Analysis

To explore the effects of soil properties on nematode abundance and diversity (including total nematodes, bacterivores, fungivores, plant parasites, and omnivore‐predators), we used the Shapiro–Wilk test to assess normality prior to significance testing and evaluated the homogeneity of variance using an *F*‐test. Differences in soil physicochemical properties along the elevational gradient were evaluated by one‐way ANOVA, followed by Tukey's HSD post hoc test (*p* < 0.05). Mixed‐effects models (LMMs) were fitted using the lmer function from the lme4 package in R. Elevation and sampling time were included as random effects in the models. Prior to model fitting, residuals were examined for normality, and log transformation was applied to nematode abundance data to correct deviations from normality. Subsequently, we quantified the proportion of variance explained by fixed effects using the *r.squaredGLMM* function from the *glmm.hp* package in R.

To assess the influence of elevation gradients on nematode community composition, we performed permutational multivariate analysis of variance (PERMANOVA) using the *adonis* function based on Bray–Curtis dissimilarity with 9999 permutations, implemented in the *vegan* package. Non‐metric multidimensional scaling (NMDS) based on Bray–Curtis dissimilarity was conducted to visualize differences in nematode community structures. Additionally, redundancy analysis (RDA) was employed to rank the explanatory power of selected soil physicochemical variables on nematode community composition.

To estimate the effects of random processes on the soil nematode community assembly, a neutral community model was used (Sloan et al. 2006). In the model, *R* ^2^ indicates the goodness of fit, which was calculated following the “Östman's method” (Östman et al. 2010). When *R* ^2^ is close to 1, the community assembly is fully consistent with random processes.

## 3 Results

### 3.1 Soil Physicochemical Properties

Soil physicochemical properties varied significantly along the elevational gradient (Figure 1). Specifically, soil moisture and NH_4_^+^‐N exhibited significant increasing trends with elevation, with the lowest values recorded at 400 m (6.34% and 7.23 mg·kg^−1^, respectively). In contrast, MBC, DOC, and AP decreased significantly along the elevational gradient, with the highest values observed at 400 m (64.38, 64.47, and 3.52 mg·kg^−1^, respectively). However, elevation had no significant effect on pH, NO_3_^−^‐N, and TP.

**FIGURE 1:**
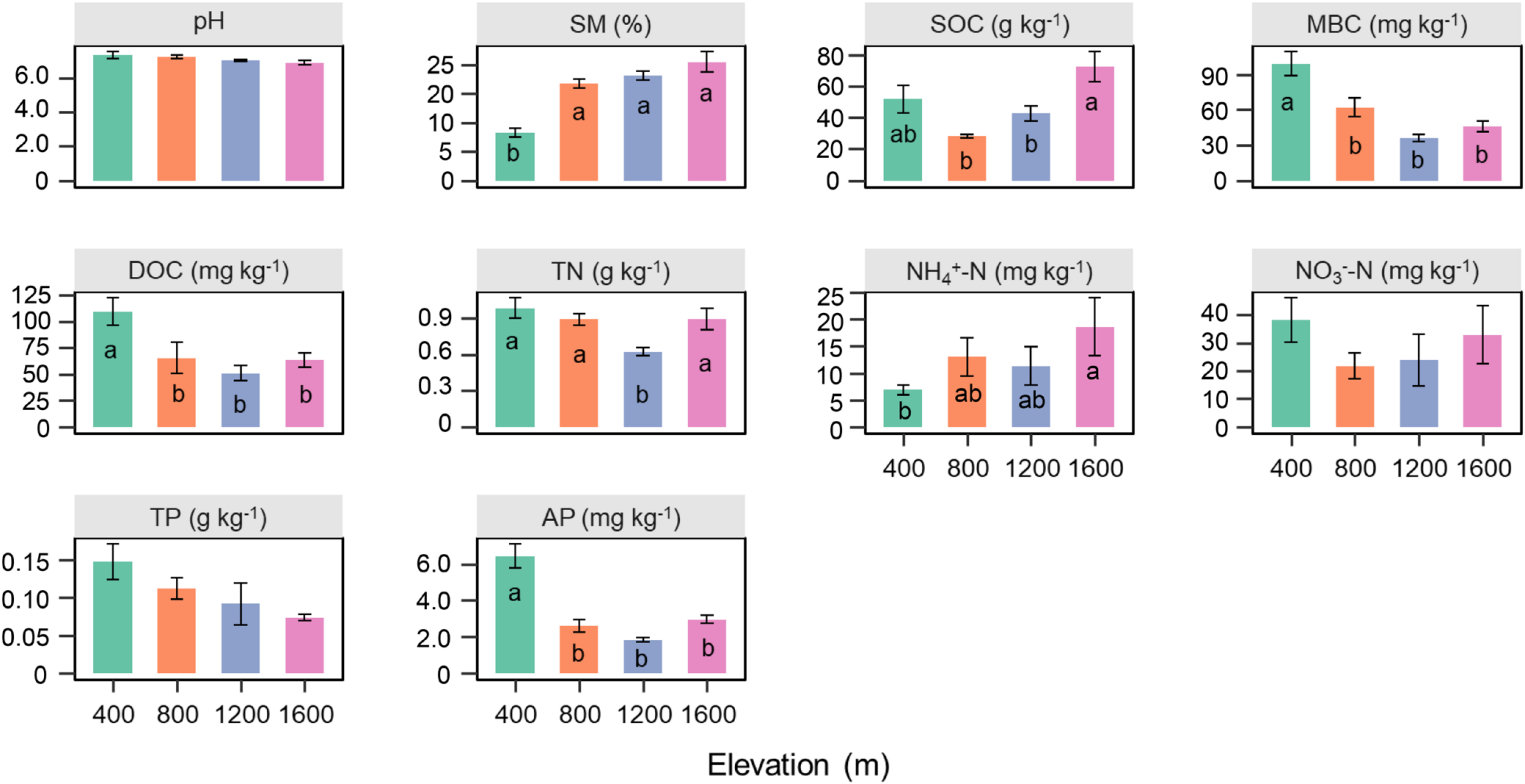
Soil properties along elevational gradients. Data are presented with standard error bars. Values are means ± SE. Different letters denote significant differences among treatments at *p* < 0.05, based on Tukey's multiple range test.

### 3.2 Effects of Soil Properties on Nematode Abundance and Diversity

Nematode total abundance and total diversity were lowest at 400 m and increased significantly with elevation (Figure 2a). Regarding trophic group abundances, BF, FF, PP, and OP all had their lowest abundances at 400 m, which were significantly lower than those at 1600 m (Figure 2b). In terms of trophic group diversity, BF diversity was highest at 400 m and significantly higher than at 1600 m, whereas PP and OP diversity were lowest at 400 m and significantly lower than at 1200 m and 1600 m (Figure 2c).

**FIGURE 2:**
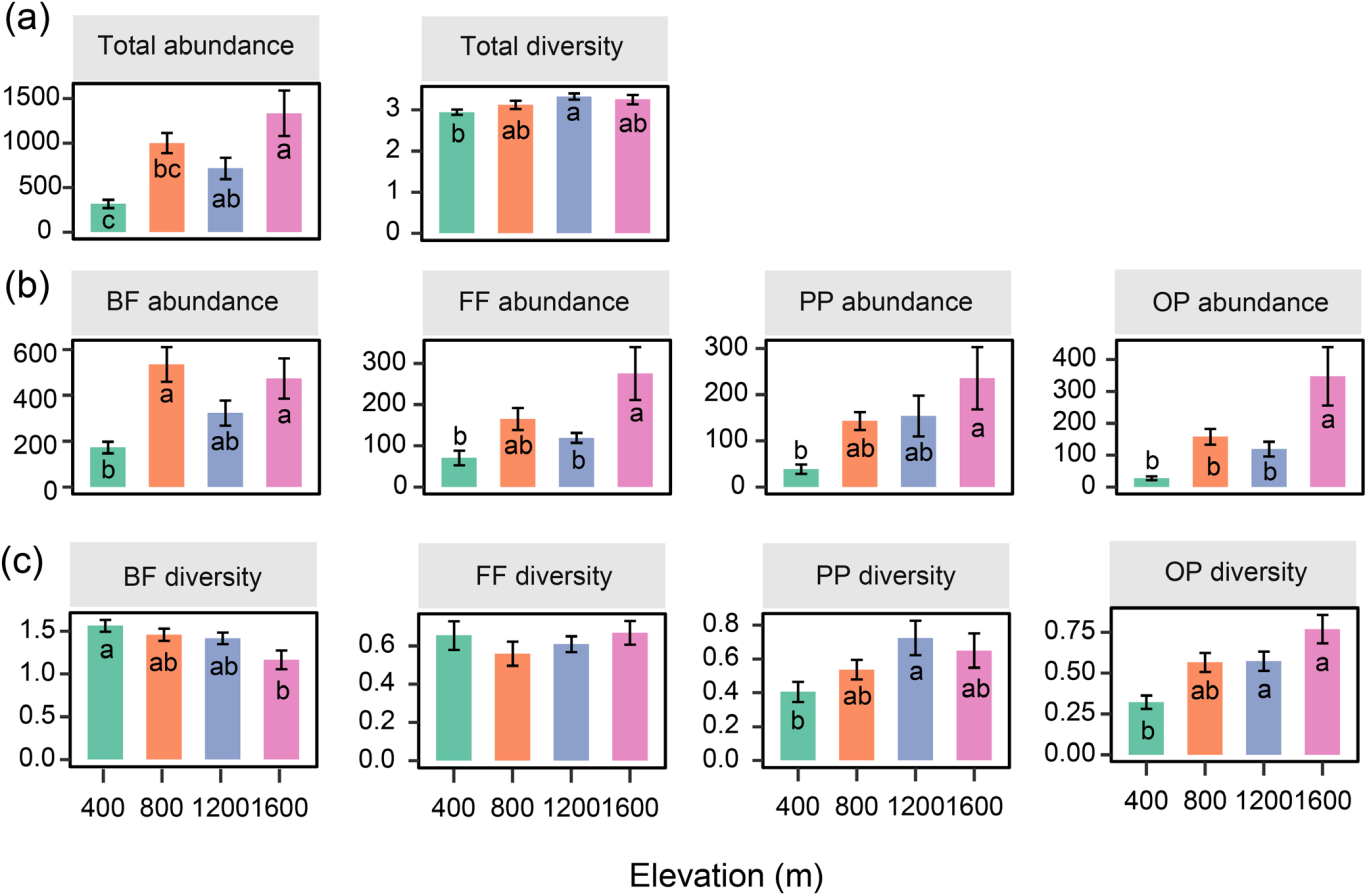
Soil nematode abundance and diversity along elevational gradients. Abundance refers to the number of nematodes per 100 g of dry soil, and diversity is expressed as the Shannon index. Bacterivores (BF), fungivores (FF), plant parasites (PP), and omnivore‐predators (OP). Values are means ± SE. Different letters denote significant differences among treatments at *p* < 0.05, based on Tukey's multiple range test.

Linear mixed‐effects models revealed that soil moisture, which increased along the elevational gradient, was significantly positively correlated with total nematode abundance, as well as with the abundances of BF, FF, and OP (Figure 3a). Furthermore, NH_4_^+^‐N was significantly positively correlated with both the abundance and diversity of PP, but negatively correlated with the diversity of BF and FF. MBC and DOC showed a significant positive correlation with BF diversity, while it was negatively correlated with the abundances of FF and PP (Figure 3a,b). AP was significantly positively correlated with BF and PP abundances. (Figure 3b).

**FIGURE 3:**
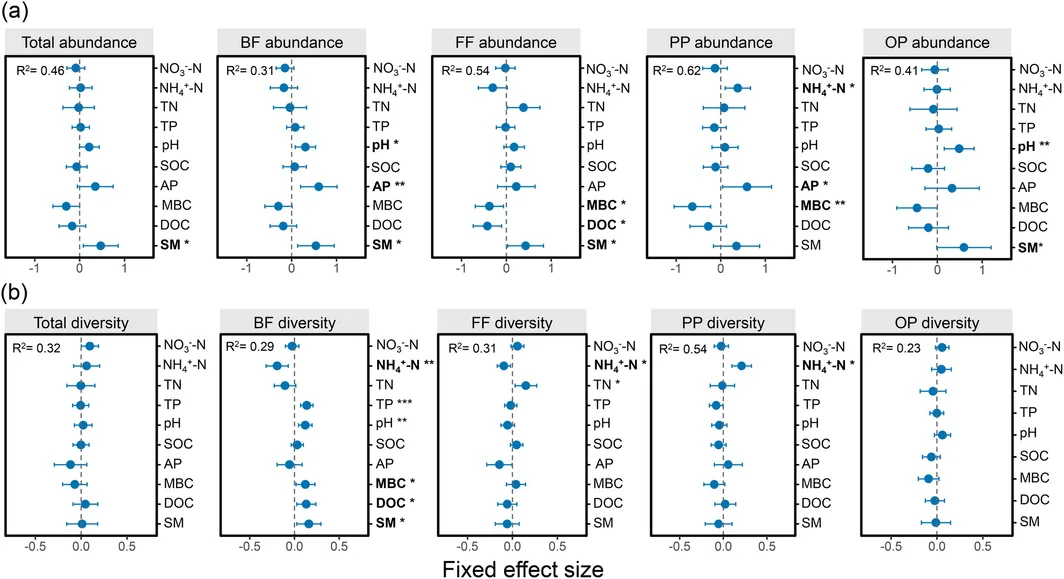
The linear mixed‐effects models illustrate the effects of soil properties on (a) log‐transformed nematode abundance (number of nematodes per 100 g dry soil) and (b) nematode diversity (Shannon diversity). Bacterivores (BF), fungivores (FF), plant parasites (PP), and omnivore‐predators (OP). Parameter significance was assessed by F‐tests (two‐tailed). R^2^ indicates the proportion of variance explained by fixed effects.

### 3.3 Nematode Community Composition Along the Elevational Gradient

Our results demonstrated that elevation significantly influenced soil nematode community composition (Adonis: *R* ^2^ = 0.209, *p* < 0.001; Figure 4a). Specifically, significant differences were observed among most elevational gradients, except between 800 m and 1200 m (Table S1). RDA was further conducted to quantitatively examine the relationships between nematode community composition and soil properties across four elevations (Figure 4b). The results revealed that MBC and NH_4_^+^‐N exerted the strongest effects on nematode community composition, explaining 62.5% and 59.3% of the variation, respectively (*p* < 0.001). In addition, AP and soil moisture also had significant influences (*p* < 0.01).

**FIGURE 4:**
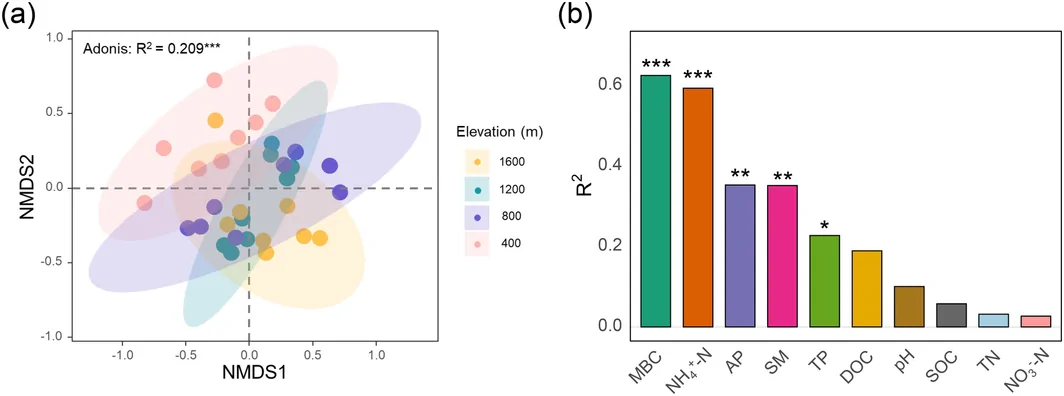
Non‐metric Multidimensional Scaling (NMDS) of nematode communities across four elevational gradients (a) and the proportion (*R* ^2^) of variation in nematode community composition explained by soil properties from Redundancy Analysis (b).

### 3.4 Nematode Community Assembly Processes Along the Elevational Gradient

The neutral community model effectively captured the relationship between nematode species' occurrence frequency and their relative abundance (Figure 5). As elevation increased, the influence of stochastic processes on nematode community variation declined, accounting for 53.2%, 41.3%, 32.6%, and 34.9% of the variance at 400, 800, 1200, and 1600 m, respectively. This indicates that the stochasticity of community assembly gradually decreased with increasing elevation.

**FIGURE 5:**
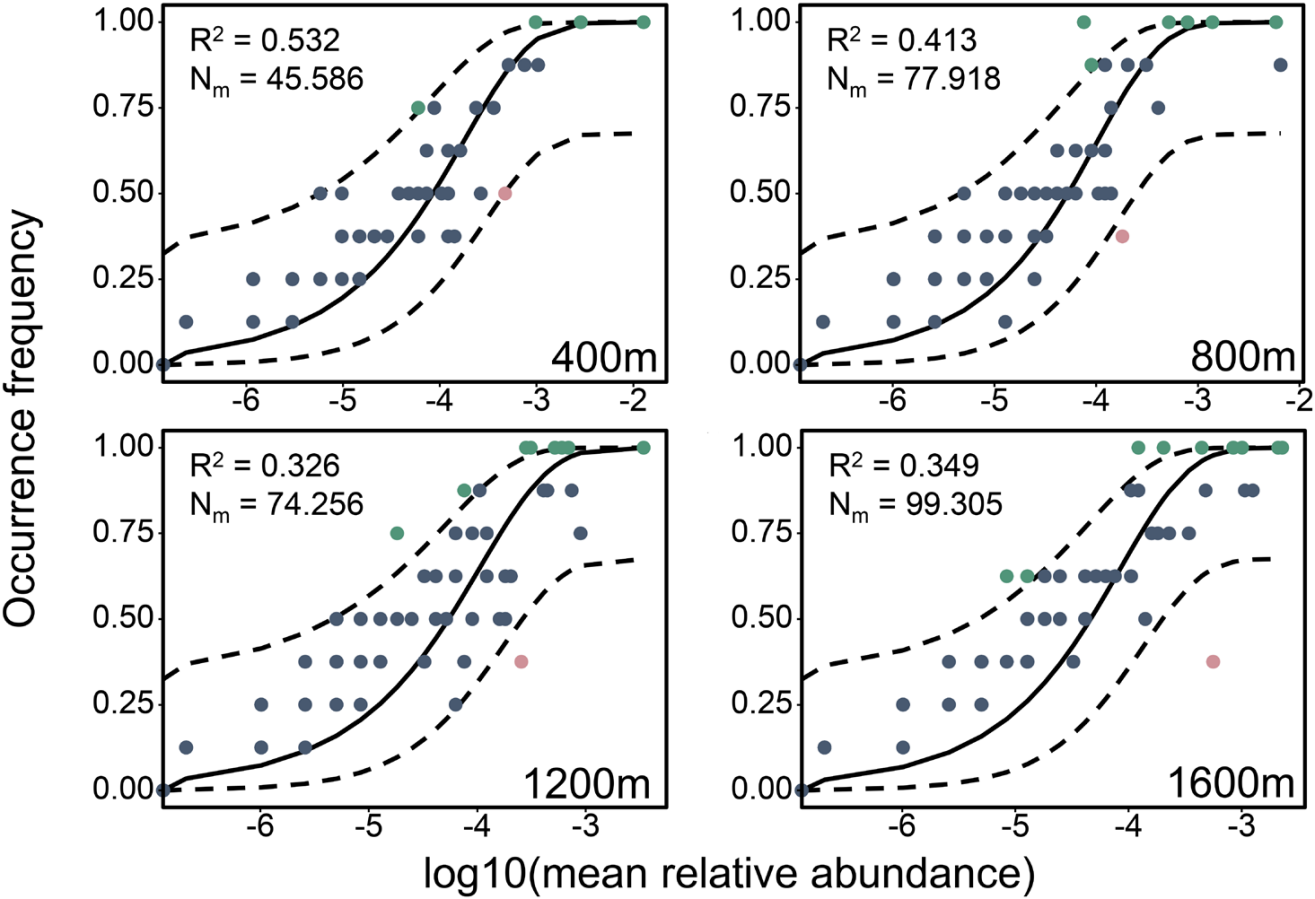
The nematode community assembly across four elevational gradients in the Yuanjiang Dry‐Hot Valley. The predicted occurrence frequencies across four elevational gradients represent nematode communities from the Yuanjiang Dry‐Hot Valley. The solid black line represents the best fit to the neutral community model (NCM), and the dashed black line indicates the 95% confidence intervals around the NCM prediction. Nematode taxa that occur more or less frequently than predicted by the NCM are shown in green and red, respectively. *R* ^2^ indicates the goodness of fit to the model.

## 4 Discussion

Our results suggest that total nematode abundance increased with elevation and higher soil moisture content, which is consistent with our hypothesis. Because nematodes inhabit the water films surrounding soil particles and rely on water for movement (Freckman et al. 1987; Nzogela et al. 2020). Thus, soil moisture is a crucial factor modulating nematode life processes (Zhang et al. 2020). Numerous studies have also reported the sensitivity of nematode abundance to soil moisture (Sylvain et al. 2014; Nielsen and Ball 2015; Xiong et al. 2020). However, the increase in soil moisture alone cannot fully explain the variation in the abundance of all trophic groups along the elevational gradient. The increases in BF and OP abundance along the elevational gradient were significantly influenced not only by soil moisture but also by soil pH and AP. In contrast, the variation in FF abundance was not only driven by soil moisture but also exhibited significant negative correlations with MBC and DOC. Taken together, these findings suggest that although soil moisture plays an important role, it offers only a partial explanation for the elevational patterns in nematode abundance.

However, contrary to our hypothesis, neither soil moisture nor nutrients could explain the elevational variation in total nematode diversity. Notably, with respect to trophic group diversity, along the elevational gradient, NH_4_^+^‐N increased the diversity of PP while decreasing the diversity of BF and FF. Nitrogen enrichment can enhance plant resources available to nematodes (Pan et al. 2016; Liu and von Wirén 2017), thereby promoting both the abundance and diversity of plant‐feeding PP nematodes (Olatunji et al. 2019). At the same time, NH_4_^+^‐N also exerts toxic effects on nematodes (Zhang et al. 2023), acting as a selective pressure that reduces nematode diversity, particularly in environmentally sensitive groups such as BF (Bongers and Marina 1998). Our results thus indicated that NH_4_^+^‐N functions dually: on one hand, it acts as a resource to promote PP abundance and diversity, and on the other hand, it serves as an environmental filter to reduce BF and FF diversity. Nevertheless, our findings could not explain the observed increase in OP diversity with elevation. This may be because OP occupy higher trophic levels, and their diversity is less directly influenced by soil physicochemical properties. A plausible explanation is that the increase in the abundance of other nematode groups along the elevational gradient may have provided OP with greater food resources (Khan and Kim 2007).

Soil moisture significantly influenced nematode community composition, which is consistent with our hypothesis. However, MBC and NH_4_^+^‐N were found to be more important drivers of nematode community composition than soil moisture. Our earlier results demonstrated that NH_4_^+^‐N reduced the diversity of BF and FF while increasing that of PP, which clearly contributed to shifts in community composition. MBC, on the other hand, is closely related to microbial metabolic activity, and nematodes—being major predators of microbes—benefit from increased microbial abundance (Jiang et al. 2017; Zhao et al. 2024). Thus, MBC can drive compositional changes in lower‐trophic nematode communities. Our findings support this, as MBC significantly increased BF diversity. Previous studies have identified phosphorus as an important factor shaping nematode community composition (Nisa et al. 2021; Ni et al. 2024). Consistently, our results also revealed significant effects of AP and TP on nematode communities. A prior study suggested that phosphorus addition might negatively affect nematode communities by reducing trophic linkages within the soil food web (Zhao et al. 2014). However, our findings instead demonstrated a positive effect of phosphorus on nematodes, as evidenced by increased abundances of BF and PP. Similarly, a recent study reported that the positive effects of phosphorus on nematode abundance may be mediated through vegetation (Liu et al. 2025). Although the mechanisms by which phosphorus regulates nematode community composition remain unclear, our findings highlight that phosphorus availability can alter nematode abundance and community structure, thereby potentially influencing nutrient cycling in soil ecosystems (Zhang et al. 2024).

The proportion of community variation explained by stochastic processes declined with increasing elevation, from 53.2% to 34.9%. This result contradicts our initial hypothesis, suggesting that the assembly of soil nematode communities in this region is not predominantly governed by environmental filtering via soil moisture. One possible explanation is that nematode communities in this arid region have already adapted to low‐moisture conditions (Bakonyi and Nagy 2000). Our study also found that the dominant nematode genus, *Acrobeles*, in the Yuanjiang Dry‐Hot Valley is typically drought‐tolerant (Table S2; (Abolafia et al. 2018)). At lower elevations, the stochasticity of nematode community assembly was higher, likely because lower soil moisture decreased nematode abundance and limited dispersal opportunities (Pequeno et al. 2021). Dispersal limitation has been shown to enhance community assembly stochasticity (Sun et al. 2021), and our results confirm this relationship between nematode population size and assembly stochasticity (Figure S1). In addition, our earlier findings demonstrated the positive effects of MBC and AP on nematode communities, as well as the selective effects of NH_4_^+^‐N. At lower elevations, MBC and AP concentrations were significantly higher, while NH_4_^+^‐N levels were lower, which reduced environmental stress on nematode communities and thereby weakened deterministic assembly processes. Previous studies have suggested that both macro‐ and micro‐ecosystems tend to exhibit stronger deterministic assembly under more extreme environmental conditions (Weiher et al. 2011; Stegen et al. 2012). Our findings indicated that in arid regions, soil moisture influences nematode community assembly indirectly by regulating nematode abundance, rather than through direct environmental filtering.

## 5 Conclusions

Our findings suggest that the increase in soil moisture is the primary driver of the elevational rise in nematode abundance, but it is not the main factor responsible for changes in nematode diversity in the Yuanjiang Dry‐Hot Valley. Instead, with increasing elevation, NH_4_^+^‐N simultaneously promoted the abundance and diversity of PP while reducing the diversity of BF. We also found that NH_4_^+^‐N and MBC exerted stronger influences on nematode community composition than soil moisture, underscoring the importance of nutrient‐related factors in shaping belowground biodiversity. Furthermore, the stochasticity of nematode community assembly decreased with elevation, suggesting that nematode communities in this arid region are adapted to low‐moisture conditions. At lower elevations, dispersal limitation and reduced environmental stress contributed to stronger stochastic processes, whereas deterministic processes became more prominent at higher elevations.

## Author Contributions

**Jinxiu Zhang:** data curation (equal), formal analysis (equal), investigation (equal), methodology (equal), validation (equal), visualization (equal), writing – original draft (equal), writing – review and editing (equal). **Haoran Lei:** formal analysis (equal), methodology (equal), visualization (equal), writing – original draft (equal), writing – review and editing (equal). **Na Lin:** data curation (equal), methodology (equal), writing – review and editing (equal). **Chunyu Hou:** data curation (equal), investigation (equal), resources (equal). **Chunyu Yue:** data curation (equal), investigation (equal), visualization (equal). **Yajun Chen:** supervision (equal), validation (equal), writing – review and editing (equal). **Jianping Wu:** funding acquisition (equal), investigation (equal), project administration (equal), supervision (equal), writing – review and editing (equal).

## Funding

This work was supported by the National Natural Science Foundation of China, 32371733. Key R&D Program of Yunnan Province, 202603AC100002. Basic Research Key Project of Yunnan Province, 202601AS070049. Project for Talent and Platform of Yunnan Province, 202605AM340003.

## Conflicts of Interest

The authors declare no conflicts of interest.

## Supporting information


**Table S1:** Pairwise PERMANOVA comparisons between nematode communities at different elevations (adjusted *p*‐values).
**Table S2:** Dominant and subdominant nematode species (highest and second highest in abundance) in soil communities across different elevational gradients in the Yuanjiang Dry‐Hot Valley.
**Figure S1:** The relationship between nematode abundance and the proportion of community variation explained by stochastic processes.

**Data S1:** ece374358‐sup‐0002‐DataS1.xlsx.

**Data S2:** Code R.

## Data Availability

All the required data are uploaded as Supporting Information.
