# Supplementary material for: Distribution Patterns and Community Assembly of Soil Nematodes Along Elevation Gradients in a Dry‐Hot Valley
Source: Ecol Evol. 2026 Sep 30;16(10):e74358. doi: 10.1002/ece3.74358 (PMC13625964; doi:10.1002/ece3.74358)
Supplement: Supplementary file 1 [file ECE3-16-e74358-s002.pdf]

# --------------------Build linear mixed-effects models---------------------------

library(sjstats)   
library(lmerTest)       
library(vegan)          
library(ggplot2)       
library(glmm.hp)        
library(lme4)  
library(dplyr)      
library(magrittr)   
library(grid)
# Data standardization
cols_to_scale <- c("SM", "DOC", "MBC", "AP", "SOC", "pH", "TP", "TN", "NH4N", "NO3N")

Raw data[cols_to_scale] <- lapply(Raw data[cols_to_scale], scale)

# Calculation of factors affecting nematode abundance; elevation as a random effect
number_model<- lmer(log(number) ~ SM + DOC + MBC + AP+ SOC + pH + TP + TN  + NH4N + NO3N +  (1 | AL)   , data = 混合效应模型)
summary(number_model)

Ba_model <- lmer(log(Ba) ~ SM + DOC + MBC  + SOC  + AP + pH + TP + TN  + NH4N + NO3N  +  (1 | AL)  , data = 混合效应模型)
summary(Ba_model)


Fu_model <- lmer(log(Fu) ~ SM + DOC  + MBC + SOC + AP + pH + TP + TN  + NH4N + NO3N   +  (1 | AL) , data = 混合效应模型)
summary(Fu_model)

Pl_model <- lmer(log(Pl) ~ SM + DOC  + MBC +  AP + SOC + pH + TP + TN  + NH4N + NO3N  +  (1 | AL)   , data = 混合效应模型)
summary(Pl_model)



OP_model <- lmer(log(OP) ~ SM + DOC  + MBC  + AP +SOC + pH + TP + TN  + NH4N + NO3N   +  (1 | AL) , data = 混合效应模型)
summary(OP_model )

# Calculation of factors affecting nematode diversity; elevation as a random effect
richness_model<- lmer(richness ~ SM + DOC  + MBC  + AP +SOC + pH + TP + TN  + NH4N + NO3N +  (1 | AL)   , data = 混合效应模型)
summary(richness_model)

BaR_model <- lmer(BaR ~ SM + DOC  + MBC  + AP +SOC + pH + TP + TN  + NH4N + NO3N  +  (1 | AL)  , data = 混合效应模型)
summary(BaR_model)


FuR_model <- lmer(FuR ~ SM + DOC  + MBC  + AP +SOC + pH + TP + TN  + NH4N + NO3N  +  (1 | AL)  , data = 混合效应模型)
summary(FuR_model)

PlR_model <- lmer(PlR ~ SM + DOC  + MBC  + AP +SOC + pH + TP + TN  + NH4N + NO3N   +  (1 | AL)  , data = 混合效应模型)
summary(PlR_model)



OPR_model <- lmer(OPR ~ SM + DOC  + MBC + SOC + pH + TP + TN  + NH4N + NO3N  +  (1 | AL)  , data = 混合效应模型)
summary(OPR_model )

# Visualization of mixed-effects model results, Ba_model as an example

P <- summary(Ba_model)  # Save model summary results
fixed_effects <- as.data.frame(P$coefficients) %>%  
  mutate(Variable = rownames(.),  
         # Extract variable names         
         Lower = Estimate - 1.96 * `Std. Error`,  # Calculate lower bound of confidence interval         
         Upper = Estimate + 1.96 * `Std. Error`,  # Calculate upper bound of confidence interval         
         Significance = case_when(  # Add significance labels           
           `Pr(>|t|)` < 0.001 ~ "***",          
           `Pr(>|t|)` < 0.01 ~ "**",          
           `Pr(>|t|)` < 0.05 ~ "*",          
           `Pr(>|t|)` < 0.1 ~ ".",        
           TRUE ~ " "         )) %>%  filter(Variable != "(Intercept)")  # Remove intercept

# Dynamically generate variable order for forest plot
forest_order <- rownames(P$coefficients) 
# Extract variable names from model results
forest_order <- forest_order[forest_order != "(Intercept)"]  
# Remove intercept
fixed_effects <- fixed_effects %>%  
  mutate(Variable = factor(Variable, levels = forest_order))  # Apply dynamically generated order


Ba <- ggplot(fixed_effects, aes(x = Estimate, y = Variable)) +
  geom_point(size = 3, color = "#0072B2") +
  geom_errorbarh(aes(xmin = Lower, xmax = Upper), height = 0.2, color = "#0072B2") +
  geom_vline(xintercept = 0, linetype = "dashed", color = "gray40") +
  scale_x_continuous(limits = c(-1.5, 1.5), breaks = seq(-2, 2, by = 1)) +
  scale_y_discrete(labels = paste0(fixed_effects$Variable, " ", fixed_effects$Significance),
                   position = "right") +
  theme_classic(base_size = 14) +
  theme(
    axis.text.y.right = element_text(size = 12, color = "black", face = "plain"),
    axis.ticks.y.right = element_line(color = "black"),
    axis.line.y.right = element_blank(),
    
    axis.text.y = element_blank(),
    axis.ticks.y = element_blank(),
    
    panel.border = element_rect(color = "black", fill = NA, linewidth = 1),
    axis.line.x = element_line(color = "black"),
    plot.margin = unit(c(1, 6, 1, 6), "lines"),
    
    panel.grid.major = element_blank(),
    panel.grid.minor = element_blank(),
    
    legend.position = "none"
  ) +
  labs(
    x = "Coefficient Estimate (95% CI)",
    y = NULL,
    title = "Fixed Effects from Mixed-Effects Model"
  )

Ba

# Export files


# Calculate model explanatory power
r.squaredGLMM(number_model)
r.squaredGLMM(Ba_model)
r.squaredGLMM(Fu_model)
r.squaredGLMM(Pl_model)
r.squaredGLMM(OP_model)
r.squaredGLMM(richness_model)
r.squaredGLMM(BaR_model)
r.squaredGLMM(FuR_model)
r.squaredGLMM(PlR_model)
r.squaredGLMM(OPR_model)



# --------------------NMDS analysis---------------------------
library(vegan)
library(ggplot2)
library(pairwiseAdonis)
# Read data
otu <- read.csv(file.choose(), head = TRUE, row.names=1)

# Grouping file
group <-  read.csv(file.choose(), header = FALSE, 
                   colClasses=c("character")) # grouping criteria
# Modify column names
colnames(group) <- c("group","samples")

# Calculate distance
otu.distance <- vegdist(otu, method = 'bray')

# PERMANOVA test followed by multiple comparisons
adonis2(otu ~ group, data = group, permutations = 999, method="bray")
# Multiple comparisons
res <- pairwise.adonis2(
  otu.distance ~ group,   # grouping variable
  data = group,
  permutations = 999,
  p.adjust.m = "fdr"             # multiple comparison correction method
)
# Output results
write.csv(res, file = "res.csv")

# NMDS ordination analysis
df_nmds <- metaMDS(otu.distance, k = 2)
# View results—focus on stress, points, and species
summary(df_nmds)

# Stress value (<= 0.2 is acceptable)
df_nmds_stress <- df_nmds$stress
df_nmds_stress

# Check the relationship between observed dissimilarities and ordination distances
# If no points are far from the line, the data are suitable for NMDS analysis
stressplot(df_nmds)

# Extract plotting data
df_points <- as.data.frame(df_nmds$points)
# Add samples variable
df_points$samples <- row.names(df_points)
# Modify column names
names(df_points)[1:2] <- c('NMDS1', 'NMDS2')
head(df_points)

p <- ggplot(df_points,aes(x=NMDS1, y=NMDS2))+
  
  geom_point(size=3)+
  
  theme_bw()
p

# Merge plotting data with grouping information
df <- merge(df_points,group,by="samples")
df$SM <- as.numeric(trimws(df$SM))
head(df)

# Visualization
color=c("#1597A5","#FFC24B","#FEB3AE","#6A5ACD")
p1<-ggplot(data=df,aes(x=NMDS1,y=NMDS2))+
  
  theme_bw()+
  
  geom_point(aes(color = group, size = SM), shape = 19)+
  
  theme(panel.grid = element_blank())+  
  geom_vline(xintercept = 0,
             lty="dashed", size = 1, color = 'grey50')+  
  geom_hline(yintercept = 0,lty="dashed", size = 1, color = 'grey50')+
  
  geom_text(aes(label=samples, y=NMDS2+0.03,x=NMDS1+0.03,vjust=0, color = group),size=3.5,
            show.legend = F)+
  
  stat_ellipse(data=df, geom = "polygon",level=0.95,linetype = 2,size=0.2,aes(fill=group),
               alpha=0.2)+  
  scale_color_manual(values = color) +
  
  scale_fill_manual(values = color)+
  
  theme(axis.title.x=element_text(size=12),
        
        axis.title.y=element_text(size=12,angle=90),
        
        axis.text.y=element_text(size=10),
        
        axis.text.x=element_text(size=10),
        
        panel.grid=element_blank())+
  
  ggtitle(paste('Stress=',round(df_nmds_stress, 3)))


# --------------------RDA analysis---------------------------
library(vegan)
library(ggrepel)
library(ggplot2)
library(ggpubr)
# Read data: nematode community data, environmental variables, and grouping information
sampledata <- read.csv(file.choose(), head = TRUE, row.names=1) # OTU data
env <- read.csv(file.choose(), header=TRUE, row.names=1)        # environmental factors
group <-  read.csv(file.choose(), header = FALSE, 
                   colClasses=c("character"))                   # grouping criteria
sampledata <- decostand(sampledata,method = "hellinger")
group <- as.list(group)

# Define fill colors for groups
col <- c("#F8766D", "#7CAE00", "#00BFC4", "#C77CFF")

# RDA analysis		
rda <- rda(sampledata, env, scale = TRUE)
rdascore <- scores(rda)
rdascore$sites
rda$CCA$biplot
rdascore$species
#write.csv(rdascore$sites,file="rda.sample.csv")
#write.csv(rda$CCA$biplot,file="rda.env.csv")
#write.csv(rdascore$species,file="rda.species.csv")

RDAE <- as.data.frame(rda$CCA$biplot[,1:2])

RDAS1 <- rdascore$sites[,1]*0.2
RDAS2 <- rdascore$sites[,2]*0.2

plotdata <- data.frame(rownames(rdascore$sites), RDAS1, RDAS2, group$V2)
colnames(plotdata) <- c("sample","RDAS1","RDAS2","group")

rda1 <- round(rda$CCA$eig[1]/sum(rda$CCA$eig)*100,2)
rda2 <- round(rda$CCA$eig[2]/sum(rda$CCA$eig)*100,2)

# RDA plot  
PP <-ggplot(plotdata, aes(RDAS1, RDAS2)) +
  geom_point(aes(fill = group, color = group),size = 5) + 
  scale_fill_manual(values = col)+
  stat_chull(geom = "polygon", aes(group = group, color = group, fill = group), alpha = 0.1)+ 
  xlab(paste("RDA1 ( ",rda1,"%"," )", sep = "")) + 
  ylab(paste("RDA2 ( ",rda2,"%"," )", sep = "")) +
  geom_segment(data = RDAE, aes(x = 0, y = 0, xend = RDAE[,1], yend = RDAE[,2]),
               colour = "black", size = 0.8,
               arrow = arrow(angle = 30, length = unit(0.4, "cm"))) +
  geom_text_repel(data = RDAE, segment.colour = "black",
                  aes(x = RDAE[,1], y = RDAE[,2], label = rownames(RDAE)),size=8) +
  geom_vline(aes(xintercept = 0), linetype = "dotted") +
  geom_hline(aes(yintercept = 0), linetype = "dotted") +
  theme(panel.background = element_rect(fill = "white", colour = "black"), 
        panel.grid = element_blank(),
        axis.title = element_text(color = "black", size = 18),
        axis.ticks.length = unit(0.4,"lines"),
        axis.ticks = element_line(color = "black"),
        axis.line = element_line(colour = "black"),
        axis.title.x = element_text(colour = "black", size = 18),
        axis.title.y = element_text(colour="black", size = 18),
        axis.text = element_text(colour = "black", size = 18),
        legend.title = element_blank(),
        legend.text = element_text(size = 18), legend.key = element_blank(),
        plot.title = element_text(size = 22, colour = "black", 
                                  face = "bold", hjust = 0.5)) +
  theme(text=element_text(family="A",size=20))
print(PP)

# Permutation test       
envfit <- envfit(rda,env,permutations  = 999)  
r <- as.matrix(envfit$vectors$r) 
p <- as.matrix(envfit$vectors$pvals)
env.p <- cbind(r,p)
colnames(env.p) <- c("r2","p-value")
K <- as.data.frame(env.p)
K
write.csv(as.data.frame(env.p), file = "RDAaenvfit.csv")


# --------------------Neutral community assembly---------------------------
library(devtools)
library(ggplot2)
library(ggimage)
library(plyr)
library(remotes)

library(MicEco)

# Read data
read_table <- read.csv(file.choose(), head = TRUE, row.names=1)

res <- neutral.fit((read_table))

m <- res[[1]][1]
N <- res[[1]][4]
Nm<- N*m
r2 <- res[[1]][3]
out <- res[[2]]

# Process data
out$group <- with(out, ifelse(freq < Lower, "#509579",
                              ifelse(freq > Upper, "#cf9198", "#485970")))

# Plot model results
p1 <- ggplot(data = out) +
  geom_line(aes(x = log(p), y = freq.pred), size = 1.2, linetype = 1) +
  geom_line(aes(x = log(p), y = Lower), size = 1.2, linetype = 2) +
  geom_line(aes(x = log(p), y = Upper), size = 1.2, linetype = 2) +
  geom_point(aes(x = log(p), y = freq, color = group), size = 3) +
  xlab("log10(mean relative abundance)") +
  ylab("Occurrence frequency") +
  scale_colour_manual(values = c("#485970", "#cf9198", "#509579")) +  # Apply the new color scheme
  annotate("text", x = -4, y = 0.10, label = paste("Nm = ", round(Nm, 3)), size = 7) +
  annotate("text", x = -4, y = 0.20, label = paste("R² = ", round(r2, 3)), size = 7) +
  theme(
    panel.background = element_blank(),
    panel.grid = element_blank(),
    axis.line.x = element_line(size = 0.5, colour = "black"),
    axis.line.y = element_line(size = 0.5, colour = "black"),
    axis.ticks = element_line(color = "black"),
    axis.text = element_text(color = "black", size = 18),
    legend.position = "none",
    legend.background = element_blank(),
    legend.key = element_blank(),
    legend.text = element_text(size = 18),
    text = element_text(family = "sans", size = 18),
    panel.border = element_rect(color = "black", fill = NA, size = 1)  # Add a panel border
  )

p1
