# Supplementary material for: Distribution Patterns and Community Assembly of Soil Nematodes Along Elevation Gradients in a Dry‐Hot Valley
Source: Ecol Evol. 2026 Sep 30;16(10):e74358. doi: 10.1002/ece3.74358 (PMC13625964; doi:10.1002/ece3.74358)
Supplement: Supplementary file 2 [file ECE3-16-e74358-s001.docx]

**Supplementary materials**

**Table S1.** Pairwise PERMANOVA comparisons between nematode communities at different elevations (adjusted p-values).

| Comparison | R^2^ | F | p_value |
| --- | --- | --- | --- |
| 400 m vs 800 m  400 m vs 1200 m | 0.151 | 2.486 | 0.013* |
|  | 0.161 | 2.68 | 0.001*** |
| 400 m vs 1600 m  800 m vs 1200 m | 0.203 | 3.572 | 0.001*** |
|  | 0.074 | 1.126 | 0.28 |
| 800 m vs 1600 m | 0.155 | 2.572 | 0.009** |

**Table S2.** Dominant and subdominant nematode species (highest and second highest in abundance) in soil communities across different elevational gradients in the Yuanjiang Dry-Hot Valley.

| **Elevation (m)** | **Genus** |
| --- | --- |
| **400** | ***Acrobeles*** |
|  | *Aphelenchoides* |
| **800** | ***Monhystera*** |
|  | *Acrobeles* |
| **1200** | ***Acrobeles*** |
|  | *Prismatolaimus* |
| **1600** | ***Aphelenchus*** |
|  | *Monhystera* |

**Figure S1.** The relationship between nematode abundance and the proportion of community variation explained by stochastic processes.
